# BLTSA: pseudotime prediction for single cells by branched local tangent space alignment

**DOI:** 10.1093/bioinformatics/btad054

**Published:** 2023-01-24

**Authors:** Limin Li, Yameng Zhao, Huiran Li, Shuqin Zhang

**Affiliations:** School of Mathematics and Statistics, Xi’an Jiaotong University, Xi’an 710049, China; School of Mathematics and Statistics, Xi’an Jiaotong University, Xi’an 710049, China; School of Mathematics and Statistics, Xi’an Jiaotong University, Xi’an 710049, China; School of Mathematical Sciences, Fudan University, Shanghai 200433, China

## Abstract

**Motivation:**

The development of single-cell RNA sequencing (scRNA-seq) technology makes it possible to study the cellular dynamic processes such as cell cycle and cell differentiation. Due to the difficulties in generating genuine time-series scRNA-seq data, it is of great importance to computationally infer the pseudotime of the cells along differentiation trajectory based on their gene expression patterns. The existing pseudotime prediction methods often suffer from the high level noise of single-cell data, thus it is still necessary to study the single-cell trajectory inference methods.

**Results:**

In this study, we propose a branched local tangent space alignment (BLTSA) method to infer single-cell pseudotime for multi-furcation trajectories. By assuming that single cells are sampled from a low-dimensional self-intersecting manifold, BLTSA first identifies the tip and branching cells in the trajectory based on cells’ local Euclidean neighborhoods. Local coordinates within the tangent spaces are then determined by each cell’s local neighborhood after clustering all the cells to different branches iteratively. The global coordinates for all the single cells are finally obtained by aligning the local coordinates based on the tangent spaces. We evaluate the performance of BLTSA on four simulation datasets and five real datasets. The experimental results show that BLTSA has obvious advantages over other comparison methods.

**Availability and implementation:**

R codes are available at https://github.com/LiminLi-xjtu/BLTSA.

**Supplementary information:**

Supplementary data are available at *Bioinformatics* online.

## 1 Introduction

Single-cell RNA sequencing (scRNA-seq) technology has been advancing the fundamental insights into biology. By measuring the gene expression at each single-cell level, it provides higher resolution views of gene expressions within heterogeneous cell populations, which leads to more accurate analysis of the static transcriptome-wide cell-to-cell variations within complex tissues (Kelsey *et al.*, 2017; Liu and Trapnell, 2016; Stubbington *et al.*, 2017), and the dynamic cell-state progression (Li *et al.*, 2018; Wagner *et al.*, 2018). In single-cell dynamics analysis, due to the difficulties in generating genuine time-series scRNA-seq data, the temporal information of the cells can only be extracted from the snapshot profiles of individual cells undergoing different developmental stages. This puts forward the problem of ordering the cells along a trajectory corresponding to their temporal states, which is also called cell trajectory inference or pseudotime analysis (Saelens *et al.*, 2019; Trapnell, 2015).

A large number of trajectory inference methods have been proposed in recent years (Haghverdi *et al.*, 2016; Ji and Ji, 2016; Liu *et al.*, 2017; Mondal *et al.*, 2021; Saelens *et al.*, 2019; Setty *et al.*, 2019; Shin *et al.*, 2015; Trapnell *et al.*, 2014; Welch *et al.*, 2016; Wolf *et al.*, 2019). According to the detected shapes or topologies of the trajectories, these methods can be divided into seven groups including cycle, linear, bifurcation, multi-furcation, tree, connected graph and disconnected graph (Saelens *et al.*, 2019). In this work, we focus on inferring the trajectories with multi-furcations. A thorough review of the methods for all possible types of topology is referred to (Bacher and Kendziorski, 2016; Saelens *et al.*, 2019).

One of the most popular approaches for inference of the cell developmental trajectory is Monocle (Trapnell *et al.*, 2014). The first version of Monocle constructs a minimum spanning tree (MST) on cells in a low-dimensional space, which is obtained using independent component analysis (ICA), and orders the cells via a PQ tree along its longest path. To implement this method, the path direction and the number of branches should be pre-specified by the users. A more recent version of Monocle (Qiu *et al.*, 2017) (Monocle2) applies reversed graph embedding to accurately reconstruct robust complex single-cell trajectories in a fully unsupervised manner. Monocle3 (Cao *et al.*, 2019) applies UMAP to reduce the dimensionality of the data, and then follows the main idea of Monocle2 to infer the pseudotime of the cells. Following Monocle, several methods also build MST to determine the lineage structure, such as Waterfall (Shin *et al.*, 2015), TSCAN (Ji and Ji, 2016) and Slingshot (Street *et al.*, 2018). These methods first cluster the cells in a low-dimensional space, then construct the MST on the cluster centers. The lineage is represented by piecewise lines or simultaneous principal curves, and the pseudotime is calculated by orthogonal projection onto these curves. For these methods, the clustering step should be conducted in advance, where a larger cluster number may lead to loss of some branches, while a smaller cluster number may result in spurious branches. Another type of methods use *k*-nearest neighbors (*k*NN) to construct the cell-to-cell relation graphs and infer the trajectories based on the graphs (Bendall *et al.*, 2014; Haghverdi *et al.*, 2016; Setty *et al.*, 2016; Wolf *et al.*, 2019). Wanderlust (Bendall *et al.*, 2014) transforms the data into an ensemble of randomly selected *k*NN graphs, where the temporal distance between cells is determined by the shortest path in each graph. The final linear trajectory is an average over all graphs. Wishbone (Setty *et al.*, 2016) extends Wanderlust to infer the bifurcating trajectories with the branching points identified using waypoints. Diffusion pseudotime (DPT) (Haghverdi *et al.*, 2016) uses diffusion maps for representing and organizing the single cells, and measures the cell-to-cell distance over diffusion-like random walks of the transition probability matrix constructed from a weighted *k*NN graph. PAGA (Wolf *et al.*, 2019) extends DPT for disconnected graphs to do pseudotime estimation. Palantir (Setty *et al.*, 2019) is also developed based on diffusion maps, and uses shortest paths from an early cell to span the differentiation landscape. It assigns each cell both the pseudotime and the branch probabilities to all terminal states. These methods are mainly designed for lineages with no more than two branches, and their performance depends on the constructed *k*NN graphs. Model-based methods have also been developed for detecting the branches, such as MFA (Campbell and Yau, 2017) and GPfates (Lönnberg *et al.*, 2017), which are mainly for bifurcations. Different from the methods that depend on dimensionality reduction technique, a recent method PseudoGA (Mondal *et al.*, 2021) is developed based on the expression of all genes and introduces a genetic algorithm to order cells with the assumption that gene expressions vary according to a smooth curve along the pseudotime trajectory. This method is implemented for each cell cluster and constructs the lineage from disjoint clusters using similar methods to MST, which may lead to misalignment of the branches.

In this work, we propose a manifold learning method: Branched Local Tangent Space Alignment (BLTSA) to reconstruct the cell developmental trajectory. This method makes full use of the local geometric structure of each single cell, and aligns the local information into global one to learn the trajectory. It first identifies the tip cells and branching cells from the local structure of each cell’s neighborhood. By clustering the non-branching cells using spectral clustering, and assigning the branching points to their nearest branches using shortest point-to-space distance, the local linear coordinates of each cell can be obtained. BLTSA finally assigns the single cells to the global trajectory by minimizing the difference between local coordinates and global ones, and the one-dimensional global coordinates of each cell are obtained. Compared to the existing pseudotime inference methods, BLTSA has several advantages including: (i) clear identification of the tip cells and branching cells; (ii) automatic detection of the number of branches; (iii) capability of handling both bifurcations and multi-furcations. In order to verify the accuracy and rationality of BLTSA method, we applied BLTSA method to both simulated datasets and real datasets, and compared with nine methods including DensityPath (Chen *et al.*, 2019), DPT (Haghverdi *et al.*, 2016), MFA (Campbell and Yau, 2017), Monocle3 (Cao *et al.*, 2019), Ouija (Campbell and Yau, 2019), Palantir (Setty *et al.*, 2019), PseudoGA (Mondal *et al.*, 2021), SLICER (Welch *et al.*, 2016) and Slingshot (Street *et al.*, 2018). The numerical results show that BLTSA has better performance than the compared methods.

## 2 Materials and methods

Consider the gene expression matrix $\tilde{X}\in R^{m\times N}$ for a cell population composed of *N* cells and *m* genes with the *i*th column being the gene expression level of cell *i*. We first remove the genes with zero expression across all the cells. $\tilde{X}$ is then normalized by size factor to adjust for read depth. The pseudotime inference problem is to order the cells according to their temporal states, that is, to map the high-dimensional gene expression vectors to one-dimensional space with each cell *i* corresponding to a pseudotime point $\tau_{i}\in R$, i=1,2,…,N.

To infer the pseudotime trajectory of the cells, normally the cells are first mapped into a lower-dimensional space, and the inference is conducted for the lower-dimensional representation. In this work, diffusion maps (Angerer *et al.*, 2016) is applied to reduce the dimensionality of the data with R package destiny. As a non-linear dimension reduction tool, diffusion maps are adapted to single-cell data by inclusion of the missing or uncertain values and adequate choice of kernel width. It preserves the cells’ noisy diffusion-like non-linear dynamics as a continuum, and is robust to noise. The data matrix after the dimensionality reduction step is denoted as $X=[x_{1},\ldots ,x_{N}]\in R^{D\times N}$, where $x_{i}$ represents the *D*-dimensional representation of cell *i*, D≪m.

**Fig. 1.**
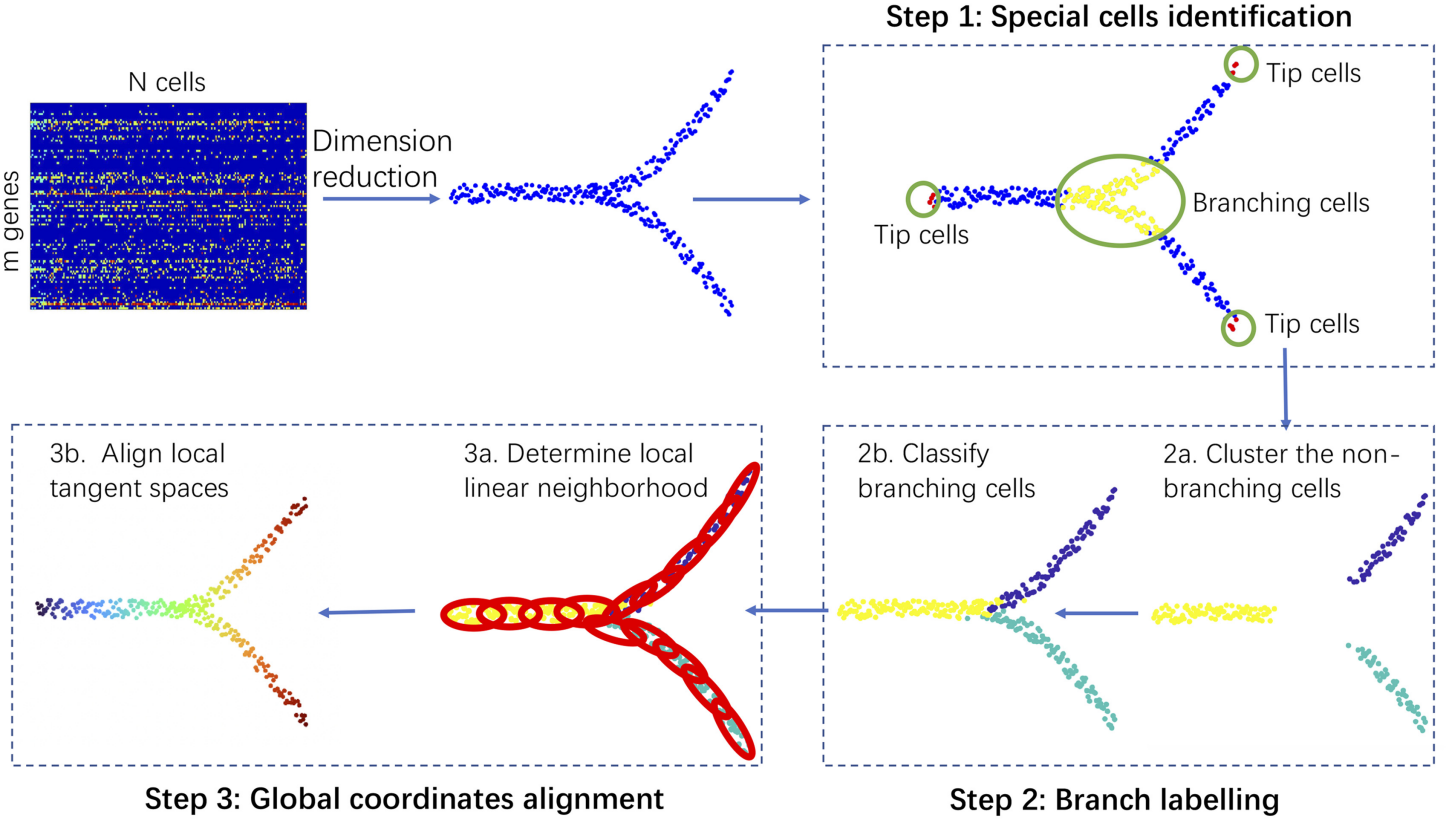
The flow chart of BLTSA. After dimension reduction, BLTSA takes three steps to obtain the pseudotime of cells. Step 1. Identifying branching cells and tip cells on the trajectory by local Euclidean neighborhoods. Step 2a. Clustering the non-branching cells to different branches. Step 2b. Classifying branching cells to different branches. Step 3a. Determining the local linear neighborhood and optimizing the local coordinates. Step 3b. Globally aligning the local coordinates to pseudotime of cells

BLTSA is developed based on the *D*-dimensional representation of the cells *X*. The main idea is to first construct local linear structure using tangent space for each cell, and then align each local tangent space to obtain the global coordinates for all the cells. Figure 1 shows the flow chart of BLTSA. It mainly includes three steps: identifying the branching cells and tip cells, clustering all the cells into different branches, determining the local coordinates of each cell and aligning all the local tangent spaces to get the global ordering of the cells.

### 2.1 Step 1: identifying the branching cells and tip cells

Cells along a differentiation trajectory can be divided into three categories: tip cells, branching cells and intermediate cells. Tip cells and branching cells are shown in Figure 1. Tip cells determine the start and end of the differentiation, branching cells locate in the intersection positions of the cell differentiation trajectory, and those remaining between these two cell categories are intermediate cells.

#### 2.1.1 Identifying the branching cells

For each cell *i*, we determine its category by learning its local geometry structure. We let $N_{i}=\{i_{1},\ldots ,i_{k}\}$ represent the set of indices for cell *i’*s *k*-nearest neighbors (including *i*), which can be found by using Euclidean distance or other appropriate distances. Let $X_{i}=[x_{i_{1}},\ldots ,x_{i_{k}}]$ represent the *D*-dimensional representation of cells in $N_{i}$. If *i* is an intermediate cell or tip cell, $X_{i}$ should approximately lie in a one-dimensional subspace after centering, while if *i* is a branching cell, $X_{i}$ should lie in a *d*-dimensional subspace with d≥2. Thus we determine the category of cell *i* by learning the dimensionality of its neighborhood subspace, which is formulated as the following optimization problem:

(1) $$\begin{array}{c} \underset{U_{i}\in R^{D\times d},\Theta_{i}\in R^{d\times k}}{\min}\|X_{i}-({\bar{x}}_{i}e^{⊤}+U_{i}\Theta_{i}){\|}_{F}^{2}, \end{array}$$

where *d* is the manifold dimensionality, ${\bar{x}}_{i}$ is the average expression for the cells in this neighborhood, $e={\left(1,1,\ldots ,1\right)}^{⊤}\in R^{k\times 1}$. The columns of the optimal $U_{i}\in R^{D\times d}$ spans a *d*-dimensional subspace that approximates the tangent space at the cell *i*, and the local coordinates of the cells in the neighborhood along the local tangent space can be obtained as $\Theta_{i}=[\theta_{i_{1}},\ldots ,\theta_{i_{k}}]$.

Clearly, the optimization problem could be solved by singular value decomposition of the centered $X_{i}$: ${\bar{X}}_{i}=X_{i}(I-\frac{1}{k}ee^{⊤})=W\Sigma V$, where *I* is the identity matrix, $W\in R^{D\times k}$ and $V\in R^{k\times k}$ consist of left and right singular vectors, respectively, and the diagonal matrix Σ consists of the singular values $\sigma_{1}\geq \sigma_{2}\geq \ldots \geq \sigma_{k}$. The optimal tangent space $U_{i}$ is chosen as the first *d* left singular vectors in *W*, and the optimal $\Theta_{i}$ is given by the first *d* rows of ΣV. For each cell *i*, the degree of non-linearity in its neighborhood $X_{i}$ is measured by

(2) $$\begin{array}{c} {\text{nonlinearity}}_{i}=\frac{\sigma_{d+1}^{2}({\bar{X}}_{i})}{\sigma_{1}^{2}({\bar{X}}_{i})+\cdots +\sigma_{d}^{2}({\bar{X}}_{i})}. \end{array}$$

A small value of ${\text{nonlinearity}}_{i}$ close to zero implies there is a big gap between the *d*th and (d+1)th singular values and the corresponding neighborhood shows a strong *d*-dimensional linearity, while a large value of ${\text{nonlinearity}}_{i}$ implies a small gap between two singular values and the neighborhood shows a weak *d*-dimensional linearity. When d=1, these two cases correspond to the tip point/intermediate point, and the branching point, respectively. We pre-specify a threshold $\delta_{b}$ for all ${\text{nonlinearity}}_{i}$ to determine the branching cells. In our experiments, we set $\delta_{b}$ as the 80% upper percentile of ${\text{nonlinearity}}_{i}$’s for all cells.

#### 2.1.2 Identifying the tip cells

Both tip cells and intermediate cells approximately lie in a one-dimensional manifold, and thus have strong one-dimensional linearity in their neighborhood. The difference is that tip cells have one differentiation direction to its neighbor cells, while intermediate cells have two opposite directions. We distinguish the tip cells and intermediate cells by measuring the angles between a given cell $x_{i}$ and its neighboring cells in $N_{i}$. For a given cell *i* and two of its neighbors *p* and *q*, if ${\left(x_{p}-x_{i}\right)}^{⊤}(x_{q}-x_{i})>0$, cell *p* and cell *q* are on the same side of the cell *i*, while if ${\left(x_{p}-x_{i}\right)}^{⊤}(x_{q}-x_{i})<0$, they are on different sides of *i*. From the definition of tip cells, if *i* is a tip cell, most of its neighbor cells should locate on the same side of *i*. Thus, we define the consistency of directions for the cell *i* as

(3) $$\begin{array}{c} {\text{consistency}}_{i}=\frac{2}{k(k-1)}\sum_{p,q\in N_{i}\backslash i}\mathrm{sgn}({\left(x_{p}-x_{i}\right)}^{⊤}(x_{q}-x_{i})), \end{array}$$

where sgn(x)=1, for x≥0, and 0 otherwise.

A ${\text{consistency}}_{i}$ close to one implies that cell *i* is a tip cell, while a relatively small ${\text{consistency}}_{i}$ implies an intermediate cell. We specify a threshold $\delta_{t}$ for all ${\text{consistency}}_{i}$ to determine the tip cells, and in our experiments, we set the threshold to be $\delta_{t}=0.9$. The identification of tip cells helps discover the root and the ends of the trajectory, and is also useful for determining the cell fate. In practice, we select the tip cell that appears at the earliest stage of the real experiment as the root cell, otherwise if the information is not given, we randomly select a tip cell as the root for further analysis.

### 2.2 Step 2: clustering cells to branches

To determine the local linear coordinates, it is necessary to assign all the cells to their corresponding branches. It is often unstable to cluster the cells to branches directly, thus we take a strategy of first clustering the non-branching cells into different branches, and then assigning the branching cells to these branches.

#### 2.2.1 Clustering the non-branching cells

The advantages to first cluster the non-branching cells are three-fold. One is that the non-branching cells can be clustered easily and stably since they are separated by the branching cells very well. The second is that the number of branches can be discovered by non-branching cells, or more accurately, tip cells. The third is that the non-branching cells have well fitted tangent space, which can be used to leverage the assignment of branching cells into different branches.

Classic clustering methods such as standard spectral clustering can be used to cluster the non-branching cells. Specifically, spectral clustering is implemented by using a symmetric normalized Laplacian matrix, where the adjacency matrix is constructed using Gaussian similarities with the standard deviation chosen as the median of the pairwise distances. The number of branches is obtained by evaluating the criterion of silhouette values. Since any non-branching cell *i* can be fitted locally linearly, and its local tangent space spanned by columns of $U_{i}$ can be well approximated by its neighborhood $N_{i}$, the non-branching cells can be used as anchor cells to extend each branch by adding branching cells iteratively using the following method.

#### 2.2.2 Assigning branching cells to different branches

Given a branching cell *i*, a number of cells closest to *i* on any branch $B_{l}$ could be first determined, the index set of which is denoted as $B_{l,i}\subset B_{l}$ with $B_{l}$ being the index set of branch $B_{l}$. The tangent space of cell $j\in B_{l,i}$ is spanned by the columns of $U_{j}$, which is approximated by its neighborhood $X_{j}$. A branching cell *i* will be assigned to a branch $B_{l}$, if the distance from the cell *i* to $B_{l}$ is the shortest over all *l’*s. The tangent distance from branching cell *i* to the branch $B_{l}$ is defined as its average distance to the tangent space of cells $j\in B_{l,i}$:

$$\begin{array}{c} d_{t}(i,B_{l})=\sum_{j\in B_{l,i}}\|(I-U_{j}U_{j}^{⊤})(x_{i}-x_{j}){\|}_{2}/|B_{l,i}|. \end{array}$$

Alternatively, we could also compute the average Euclidean distance from branching cell *i* to the branch $B_{l}$ as

$$\begin{array}{c} d_{e}(i,B_{l})=\sum_{j\in B_{l,i}}\|x_{i}-x_{j}{\|}_{2}/|B_{l,i}|. \end{array}$$

In practice, we take the average of tangent distance and Euclidean distance as the distance between cell *i* and branch $B_{l}$. By varying *l*, we can compute the distance between cell *i* and branch $B_{l}$ for all branches. Then we add cell *i* to its closest branch $B_{l}$, and delete *i* from the branching cells. Furthermore, the neighborhood of cell *i* is updated as the cells both in original $N_{i}$ and $B_{l}$, and the corresponding tangent space is updated as well. This step is repeated until all the branching cells are assigned. With the iterative procedure, the original branches consisting of the non-branching cells only are extended by the branching cells.

### 2.3 Step 3: globally aligning local neighborhoods

Once each cell is assigned to one of the branches by the above iterative procedure, its neighbors are redistributed such that they are from either the same branch or the root branch.

The redistributed neighborhood for the cell *i*, for simplicity, still denoted as $N_{i}=\{i_{1},\ldots ,i_{k}\}$ for cell indices and $X_{i}$ for gene expression, could be used to determine the local coordinates $\Theta_{i}=[\theta_{i_{1}},\ldots ,\theta_{i_{k}}]\in R^{d\times k}$ by Equation (1). Here an optimal neighborhood size *k* is chosen adaptively using the method proposed in (Zhang *et al.*, 2012), where only a range of the neighborhood size [$k_{\text{min}},k_{\text{max}}$] is needed in practice.

We then align the local coordinates $\left\{\Theta_{i},i=1,2,\ldots ,N\right\}$ over all neighborhoods to global coordinates $\left[\tau_{1},\tau_{2},\ldots ,\tau_{N}\right]$ for all the cells, borrowing the idea of the method in (Zhang and Zha, 2004). Denote the unknown global coordinates for all the cells as a matrix $T=[\tau_{1},\ldots ,\tau_{N}]$, and then the global coordinates for *k* neighbors of the cell $x_{i}$ are $[\tau_{i_{1}},\ldots ,\tau_{i_{k}}]=T_{i}=TS_{i}\in R^{d\times k}$, where $S_{i}\in R^{n\times k}$ is the 0–1 neighborhood selection matrix for $N_{i}$.

In each neighborhood $N_{i}$, the global coordinates $T_{i}=[\tau_{i_{1}},\ldots ,\tau_{i_{k}}]$ and the local coordinates $\Theta_{i}=[\theta_{i_{1}},\ldots ,\theta_{i_{k}}]$ should satisfy

(4) $$\begin{array}{c} \tau_{i_{j}}={\bar{\tau }}_{i}+L_{i}\theta_{i_{j}}+\epsilon_{i_{j}}, \end{array}$$

for each j=1,…,k, where ${\bar{\tau }}_{i}$ is the average of $\tau_{i_{j}}$ in neighborhood $N_{i}$, $\epsilon_{i_{j}}$ is reconstruction error and $L_{i}$ is an unknown affine transformation. In matrix form, it can be written as:

$$\begin{array}{c} T_{i}=\frac{1}{k}T_{i}ee^{⊤}+L_{i}\Theta_{i}+E_{i}, \end{array}$$

where $E_{i}=[\epsilon_{i_{1}},\ldots ,\epsilon_{i_{k_{i}}}].$ For a fixed $T_{i}$, The optimal alignment matrix $L_{i}$ minimizing $E_{i}$ can be given by $L_{i}=T_{i}(I-\frac{1}{k}ee^{⊤})\Theta_{i}^{+}$, where $\Theta_{i}^{+}$ is the pseudo-inverse of $\Theta_{i}$, and thus

(5) $$\begin{array}{c} E_{i}=T_{i}(I-\frac{1}{k}ee^{⊤})(I-\Theta_{i}^{+}\Theta_{i})=T_{i}W_{i}=TS_{i}W_{i}, \end{array}$$

where $W_{i}=(I-\frac{1}{k}ee^{⊤})(I-\Theta_{i}^{+}\Theta_{i})$.

We seek to find optimal global coordinates *T* to minimize the reconstruction errors over all local neighborhoods as follows:

(6) $$\begin{array}{c} \underset{T}{\min}\sum_{i=1}^{N}\|E_{i}{\|}_{F}^{2}=\underset{T}{\min}\sum_{i=1}^{N}\|TS_{i}W_{i}{\|}_{F}^{2}=\underset{T}{\min}\|\mathrm{TSW}{\|}_{F}^{2}, \end{array}$$

where $W=\text{diag}(W_{1},\ldots ,W_{N})$, $S=[S_{1},\ldots ,S_{N}]$. To avoid trivial solutions, we impose a constraint of $T^{⊤}T=I$, and thus the optimal solution of *T* for problem (6) is given by the eigenvectors of $SWW^{⊤}S^{⊤}\in R^{N\times N}$ corresponding to the *d* smallest eigenvalues. For pseudotime inference problem, d=1, and we take the corresponding eigenvector or its opposite such that the value for the root is the smallest. Then the values of the vector are mapped to the range between 0 and 1 with 0 being the root of the trajectory. The computational complexity for eigenvalue decomposition problem in BLSTA algorithm is only $O(N^{2})$ for pseudotime inference.

The pseudo-code for the BLSTA algorithm is shown in the Supplementary Materials.

## 3 Results

In this section, we applied BLTSA to both simulated datasets and real datasets to show its performance, and compared it with the following nine trajectory inference methods: DensityPath (Chen *et al.*, 2019), DPT (Haghverdi *et al.*, 2016), MFA (Campbell and Yau, 2017), Monocle3 (Cao *et al.*, 2019), Ouija (Campbell and Yau, 2019), Palantir (Setty *et al.*, 2019), PseudoGA (Mondal *et al.*, 2021), SLICER (Welch *et al.*, 2016) and Slingshot (Street *et al.*, 2018).

When implementing BLTSA, we directly applied diffusion maps to reduce the dimensionality of the genes to two (D=2). The root cell is given beforehand or taken as the tip cell that appears at the earliest time of the real experiment for pseudotime inference. There are three parameters we need to choose in BLTSA, including the neighbor size *k* for identifying special cells and the adaptive range $\left[k_{\text{min}},k_{\text{max}}\right]$ of neighborhood size for aligning the local neighborhoods. In practice, we fixed the neighborhood size as k=50 for all the datasets except dataset Setty (k=1000), while for aligning the local neighborhoods, we set the range [$k_{\text{min}},k_{\text{max}}$]=[40,100] for adaptive neighborhood selection for almost all our experiments, except that $k_{\text{min}}=20$ for dataset Sun, $k_{\text{max}}=200$ for Simulation 4, and 400 for dataset Setty.

### 3.1 Simulation study

We simulated four datasets (two with bifurcation and two with trifurcation) with different types of cell differentiation trajectories to show the performance of BLTSA. The left column in Figure 3 visualizes the four datasets by diffusion maps. The four datasets are generated by the following procedure.

- **Simulation 1** We applied the data simulation method in MFA (Campbell and Yau, 2017) to generate 200 cells with 40 genes on a cell differentiation trajectory with bifurcation. We used the sigmoid function in (Campbell and Yau, 2017) to construct the mean, 1.5*Pois(3) to construct the standard deviation, and generated the data from a Gaussian noise model ensuring any negative values being zero.
- **Simulation 2** We generated 200 cells with 40 genes by similar procedure to that in Simulation 1. The difference is that 2/Gamma(2,2) was used to construct the standard deviation.
- **Simulation 3** This dataset includes 400 cells. The first 100 cells on the root branch were sampled from a straight line with vertical coordinates as 1 and horizontal ordinates in [0, 1]. This branch was differentiated into 3 branches with all the 3 branches having horizontal ordinates in [1, 3] with equal distance between neighboring cells. The vertical coordinates of the 3 branches followed the function $f(x)=0.75+0.25x^{1.75}$, $f(x)=1.25-0.25x^{1.75}$, and a constant value 1, respectively. Then the uniform noise with random values from 0 to 0.6 was added to the data.
- **Simulation 4** We applied the data simulation method in VeloSim (Zhang and Zhang, 2021) to generate 499 cells with 100 genes on a cell differentiation trajectory with trifurcation. We constructed a tree structure with three branching events to generate this dataset.

#### 3.1.1 Illustration of BLTSA

**Fig. 2.**
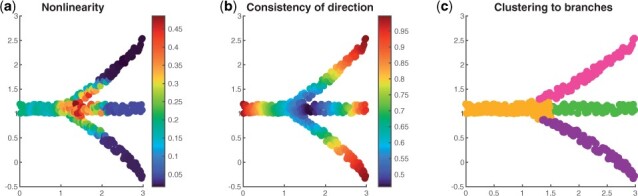
Illustration of BLTSA using Simulation 3. (**a**) Branching cell identification using ${\text{nonlinearity}}_{i}$. Color bar shows the values of ${\text{nonlinearity}}_{i}$. For the true branching cells, ${\text{nonlinearity}}_{i}$ has relatively high value. (**b**) Tip cell identification using ${\text{consistency}}_{i}$. Color bar shows the values of ${\text{consistency}}_{i}$. For the true tip cells, ${\text{consistency}}_{i}$ has relatively high value. (**c**) Identified branches. Different colors show the different branches

We illustrate the branching and tip cell identification step and assignment of cells to branches using dataset Simulation 3 in Figure 2. Figure 2a shows the branching cell identification step. For each cell, the corresponding ${\text{nonlinearity}}_{i}$ is calculated. A cell with larger value of ${\text{nonlinearity}}_{i}$ is more likely to be the branching cell. It is clear that with a proper threshold, the branching cells can be identified correctly. Figure 2b shows the tip cell identification. Tip cells should have larger values of ${\text{consistency}}_{i}$, which is consistent with what is shown in the figure. Figure 2c shows the assignment of cells into different branches. After using spectral clustering to cluster the non-branching cells, the branching cells are assigned to their nearest branch. For Simulation 3, it is clear that the four branches can be well identified.

#### 3.1.2 Single-cell differentiation trajectory inference for the simulated datasets

**Fig. 3.**
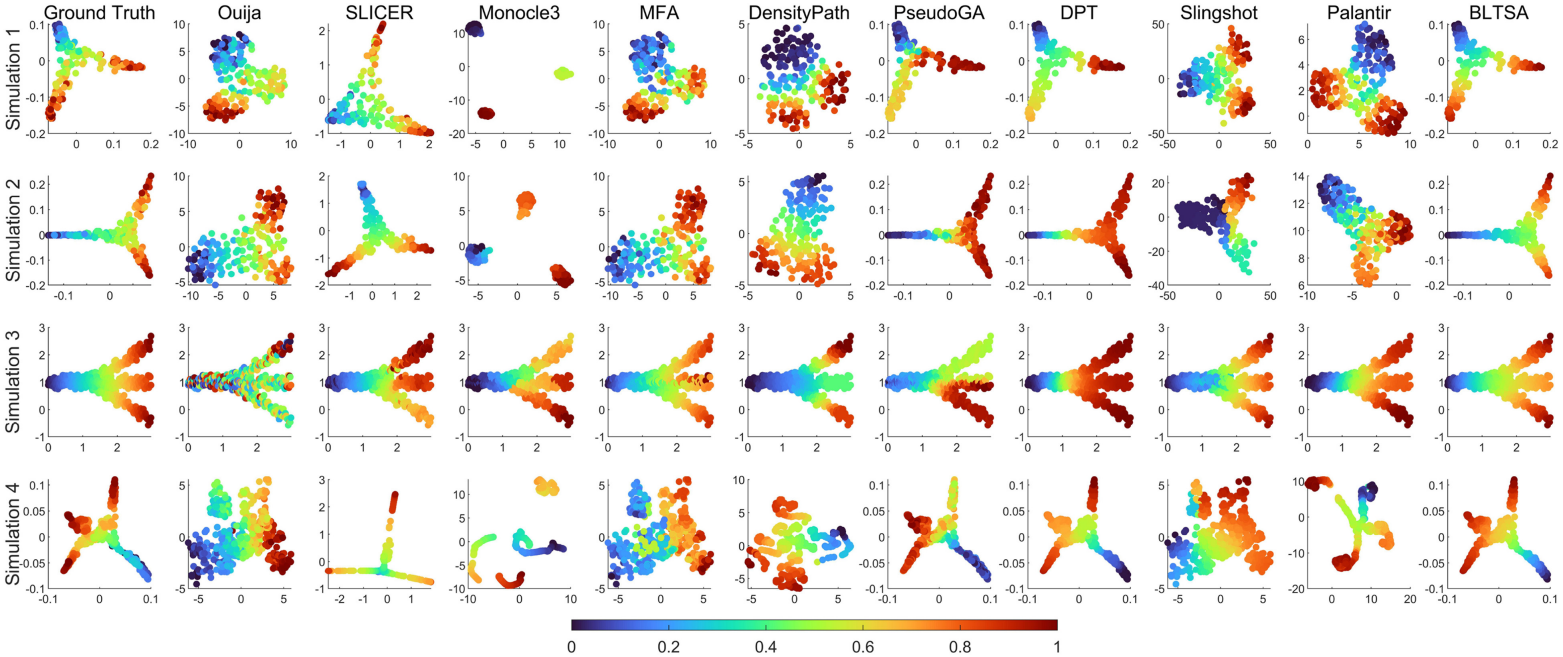
Comparison results for 10 single cell differentiation trajectory inference methods in four simulation datasets. The color bar value from small to large indicates the differentiation progress

We applied BLTSA and the compared methods to the four simulated datasets. For the compared methods, we set all the related parameters as default. The visualization of the inferred trajectories is shown in Figure 3.

For all these four datasets, BLTSA gives very clear pattern of cell development. The pseudotime values were arranged evenly according to the order of cell differentiation. While SLICER, DensityPath, DPT and Palantir perform comparably in four datasets, our BLTSA performs more robustly. Some of the remaining methods perform well for particular cell differentiation patterns, for example, Ouija and PseudoGA perform well for Simulation 2, MFA for the first three datasets, and Slingshot for Simulation 1 and 3.

To quantitatively evaluate the performance of the nine methods, we calculated the Pearson Correlation Coefficient (PCC) and the Spearman’s Rank Correlation Coefficient (SRCC) between the inferred pseudotime and the ground truth pseudotime of the cells in the simulation. The results are shown in Table 1. The higher the value, the better the method. The results show that our BLTSA obtains the best PCC in three of four datasets and the second best SRCC in three of four datasets. Although DPT could obtain similar SRCC with BLTSA, its PCCs are significantly lower than those of BLTSA. This implies that BLTSA could not only infer the order of the cells, it could also infer pseudotime more accurately. Furthermore, our BLTSA could obtain higher PCC or SRCC values than Palantir.

**Table 1.** Comparison of 10 single cell differentiation trajectory inference methods in the four simulated datasets

Metric	Method	Ouija	SLICER	Monocle3	MFA	DensityPath	PseudoGA	DPT	Slingshot	Palantir	BLTSA
PCC	Simulation 1	0.888	0.918	0.781	0.950	0.936	0.855	0.899	0.953	**0.957**	**0.957**
	Simulation 2	**0.967**	0.944	0.853	0.949	0.946	0.926	0.917	0.788	0.936	0.965
	Simulation 3	0.152	0.945	0.921	0.975	0.927	0.808	0.942	0.984	0.977	**0.987**
	Simulation 4	0.701	0.890	0.843	0.633	0.944	0.871	0.936	0.796	0.943	**0.951**
SRCC	Simulation 1	0.882	0.914	0.749	0.942	**0.957**	0.829	0.930	0.953	0.947	0.955
	Simulation 2	0.971	0.950	0.806	0.957	0.949	0.944	**0.973**	0.855	0.946	0.970
	Simulation 3	0.161	0.950	0.903	0.980	0.981	0.833	**0.996**	0.988	0.988	0.993
	Simulation 4	0.678	0.900	0.799	0.600	**0.944**	0.793	0.901	0.771	0.921	0.936

*Note*: We used Pearson Correlation Coefficient (PCC) and Spearman’s Rank Correlation Coefficient (SRCC) to measure the correlations between the inferred pseudotime and the real pseudotime in the simulations.

Bold font denotes the highest value, and underline denotes the second highest value.

### 3.2 Analysis of real biological data

#### 3.2.1 Datasets

We applied BLTSA and the other nine state-of-the-art cell differentiation trajectory inference methods mentioned above to the following five public real datasets.

- **Shalek** This dataset (Shalek *et al.*, 2014) is a single-cell RNA-seq library from primary mouse bone-marrow-derived dendritic cells, which includes 1861 cells with 27 723 genes. We screened genes significantly associated with the three major PCs in four modules (Shalek *et al.*, 2014), and chose the gene expression stimulated with lipopolysaccharide after 1, 2, 4 and 6 h as our experimental data. Finally we got 383 cells with 342 genes.
- **Sun** This dataset (Sun *et al.*, 2017) is from mouse ESCs. Dense time points were selected during neural differentiation based on qPCR results. mRNA-sequencing was performed at both cell population and single-cell levels. There are 64 cells in this dataset with 11 449 genes. We filtered out genes that were expressed in <20 cells, resulting in a dataset of 64 cells with 10 049 genes.
- **Guo** This dataset (Guo *et al.*, 2010) is single-cell qPCR data from mouse blastocysts, including 3 different cell types. The authors identified 48 genes in 442 cells based on whole-embryo analysis of more than 800 transcription factors. We screened data from the 2-cell stage to the 64-cell stage, including 433 cells containing 48 genes.
- **Petropoulos** This dataset (Petropoulos *et al.*, 2016) is of human preimplantation embryos, which contains the expression profile of 1529 single cells with 26 178 genes in RPKM values. It was labeled from embryonic day 3–7 (E3–E7). We screened cells at the E5 stage and filtered out genes that were expressed in <20 cells. Finally we got 377 cells with 15 985 genes.
- **Setty** This dataset (Setty *et al.*, 2019) is of CD34+ human bone marrow cells, containing 4142 cells and 16 106 genes. We filtered out genes that were expressed in <20 cells. In order to facilitate data dimensionality reduction, PCA was used to reduce the data to 300 dimensions. After dimension reduction, we took 90% quantile of the distances to the 100 nearest neighbors for all the cells as the threshold, and deleted those cells with <40 neighbors within this threshold. Finally, we got 4033 cells with 13 367 genes.

For all datasets except Petropoulos and Setty, we selected the tip cell that appears at the earliest stage of the real experiment as the root cell. For datasets Petropoulos and Setty, which do not have information for real experimental timepoints, we randomly select one tip cell identified by our method as the root cell. All the methods use the same root for a fair evaluation.

#### 3.2.2 Results of cell differentiation trajectory prediction

**Fig. 4.**
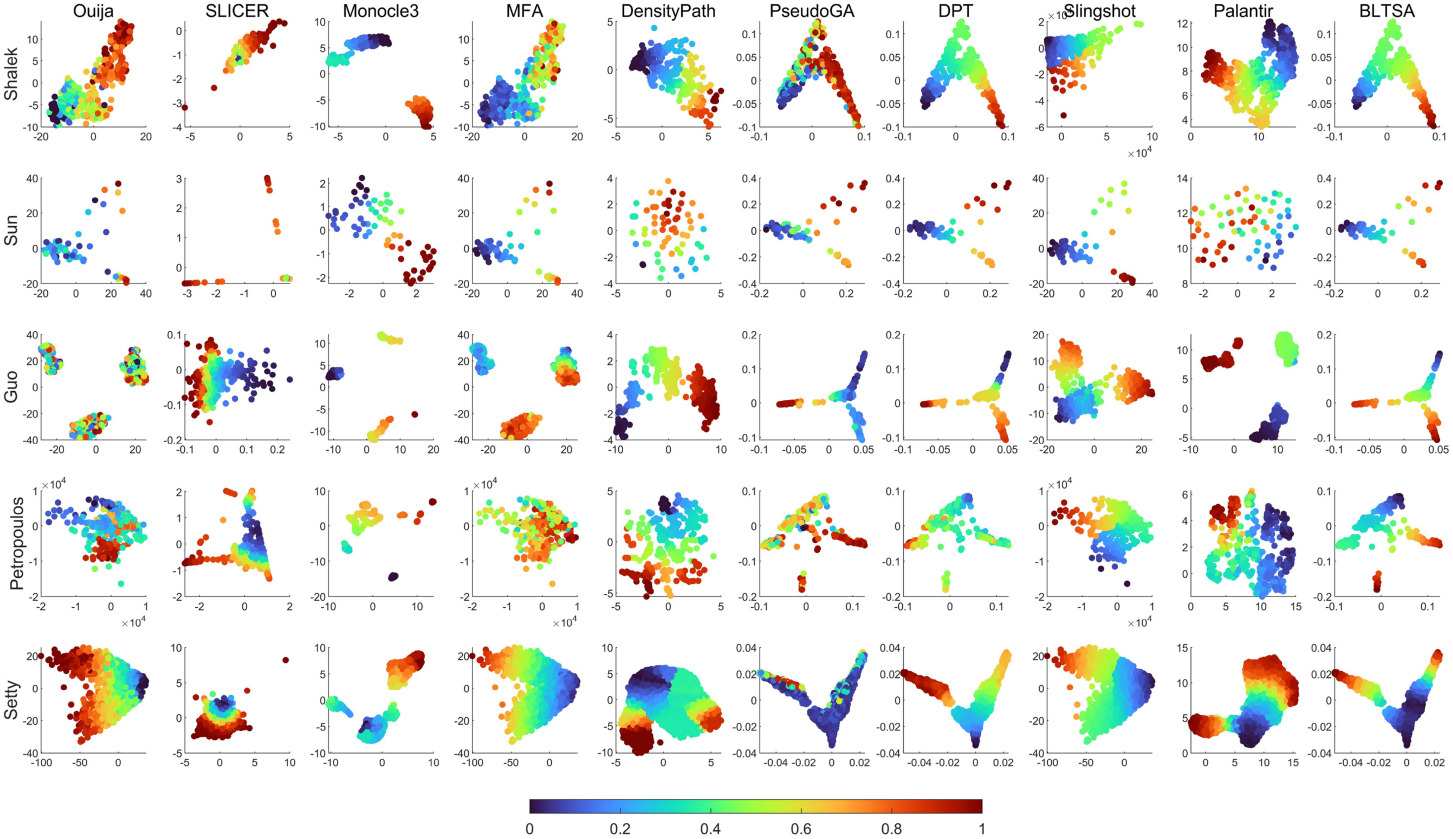
Results of cell differentiation trajectory inference by 10 methods. The color bar represents the predicted pseudotime

Figure 4 shows the visualization of cell differentiation trajectories inferred by the 10 considered methods. For the nine compared methods, we directly used the default settings. Dataset Shalek has no bifurcation; Sun and Setty have one bifurcation, while Guo and Petropoulos have one trifurcation.

The Shalek dataset has no bifurcation. The cell differentiation progress can be seen in the trajectories produced by all the 10 methods. Depending on the dimensionality reduction methods, the visualization shows different patterns. For DPT, PseudoGA and BLTSA, the data dimensionality is reduced by diffusion maps. DPT and BLTSA give similar smooth trajectories, while PseudoGA gives some noisy pattern. For Ouija and Monocle3, the cells are more likely to cluster into small groups instead of a continuous trajectory. For both DensityPath and Slingshot, though the trajectory is not smooth, the cells ordering can be observed. Palantir also gives a clear cell developmental pattern.

For the dataset Sun with bifurcation, BLTSA gives smooth and evenly distributed cell ordering. MFA, DPT, PseudoGA and Slingshot show similar patterns, though the differences of the ordering can be clearly seen. SLICER and Ouija could produce bifurcations, but the ordering has relatively low correlations with the given experimental time. For Monocle3, DensityPath and Patantir, there is no bifurcation in the inferred trajectory, though the pattern of cell development can be seen.

For the dataset Guo, BLTSA gives a very clear trajectory of cell development. The pseudotime is continuous and its increasing is very smooth. The trajectory produced by DPT and PseudoGA shows similar patterns. For Ouija, Monocle3, MFA, Slingshot and Palantir, the cells are more likely to be clustered into three groups. The transitions between the groups are obscure. For SLICER and DensityPath, the trajectory has no bifurcations, and the cells develop along one direction.

In the dataset Petropoulos, at the E5 stage, the trophectoderm (TE) and inner cell mass (ICM) segregate first, and the ICM cells were then segregated into epiblast (EPI) and primitive endoderm (PE). BLTSA can clearly identify the three branches with smooth developmental pattern. SLICER, Slingshot, DensityPath and Palantir also show a smooth developmental pattern though the branches are not clearly separated. For other methods, the cell ordering shows some random patterns.

The dataset Setty has a bifurcation with a large number of cells. Almost all the methods could show the pattern of cell development, though the patterns are not consistent. We also recorded the computational time of all methods, which is put in the Supplementary Materials.

To check the robustness of the parameters, we further did experiments on dataset Guo for different values of *k*, *k*_min_ and *k*_max_ and put the results in the Supplementary Materials.

#### 3.2.3 Analysis of the trajectories inferred by BLTSA

In this subsection, we further look at the trajectory inference results for the real datasets with known experimental time points using BLTSA.

**Fig. 5.**
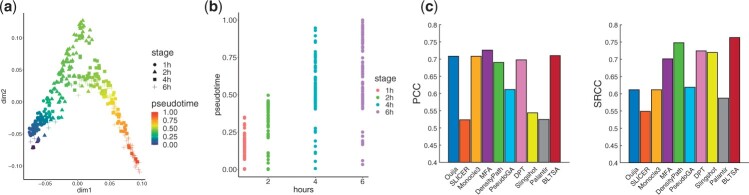
Pseudotime inference results in Dataset Shalek. (**a**) Visualization of the inferred trajectory with given corresponding time points. (**b**) Correspondence between the predicted pseudotime and the given experimental time point. (**c**) Comparison of the 10 methods between the predicted pseudotime and the known experimental time points measured using PCC and SRCC

Dataset Shalek consists of 306 dendritic cells and 74 genes in mouse. The cells were stimulated with a pathogenic component, and their gene expression changed. Figure 5a shows the predicted trajectory marked with both the real experimental time points and the predicted pseudotime. It is clear that the predicted pseudotime is highly consistent with the real experimental time after stimulation. Figure 5b further confirms the high correspondence between the predicted pseudotime and the given experimental time points. To compare the performance of all the 10 pseudotime inference methods, we calculated the PCC and SRCC between the given experimental time and the inferred pseudotime, and the result is shown in Figure 5c. BLTSA achieves the highest SRCC among all the 10 methods, and the PCC is also comparable to the best one.

**Fig. 6.**
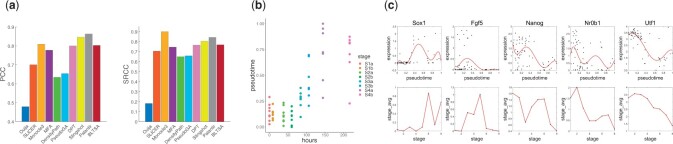
Pseudotime inference results in Dataset Sun. (**a**) Comparison of the 10 methods between the predicted pseudotime and the known experimental time points using PCC and SRCC. (**b**) Correspondence between the predicted pseudotime using BLTSA and the given experimental time point. $S_{\text{xa}}$ denotes the ‘x’ stage and time point ‘a’. (**c**) Comparison of the expression of selected development-related marker genes in inferred pseudotime and the average expression at known experimental time points. A polynomial curve is fitted for the expression ordered by pseudotime in single cell data

Dataset Sun studied the *in vitro* neural differentiation process of mouse embryonic stem cells (mESCs), which included both single-cell data and cell population data as reference. Figure 6a shows the comparison of the PCC and SRCC between the predicted pseudotime and the known experimental time point. BLTSA achieves the comparable results with DPT, Monocle3, Slingshot and Palantir according to both PCC and SRCC. The predicted pseudotime for the cells in each time point is shown in Figure 6b. It is clear that the predicted pseudotime has high correlations with the known experimental time points. To compare the gene expression development in inferred single-cell trajectory with the reference cell population, we selected a few specific genes that are marker genes of some cell developmental stage (Sox1, Fgf5) or that have high correlations with both the predicted differentiation time and the fitted differentiation time (Nanog, Nr0b1, Utf1) (Sun *et al.*, 2017). Figure 6c shows the results. We fitted the gene expression in single-cell data using a polynomial curve. For all these five genes, the gene expression in single-cell trajectory and cell populations shows high correlations, with that in single cells shows more smooth pattern. Similar patterns can be observed in other related genes.

**Fig. 7.**
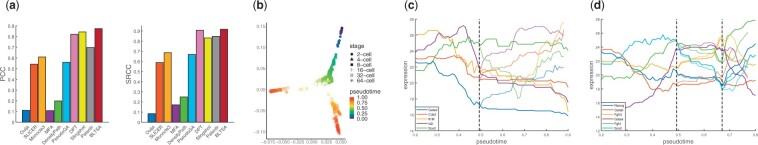
Pseudotime inference results in Dataset Guo. (**a**) Comparison of the 10 methods between the predicted pseudotime and the known experimental time points using PCC and SRCC. (**b**) Visualization of the inferred trajectory. (**c**) The solid line shows the gene expression of TE and the dotted line shows the gene expression of PE. (**d**) The solid line shows the gene expression of PE and the dotted line shows the gene expression of EPI

Dataset Guo consists of 428 cells with 48 genes. Figure 7a shows the PCC and SRCC between the inferred pseudotime and the cell developmental stages. BLTSA achieves the best PCC and the SRCC is comparable to DPT. Figure 7b shows the developmental period of the cells beginning from the 2-cell zygote to the 64-cell blastocyst. It is clear that the inferred trajectory is quite consistent with the cell developmental process. The 64-cell blastocyst is divided into 3 types including trophectoderm (TE), epiblast (EPI) and primitive endoderm (PE). The figure also shows that there are three branches at the end of the trajectory. According to the description of the dataset in (Guo *et al.*, 2010), the three types of cells: TE, EPI and PE, differentiate at the 64-cell stage, where TE first forms at approximately the 32-cell stage and EPI and PE are formed by the inner cell mass (ICM). After sorting the gene expression data according to the inferred pseudotime, we replaced the gene expression value at each point with the average of its 10 nearest neighbors before and after to plot the gene expression curve in order to smooth the original gene expression curve and facilitate the observation of trends. The black dashed line indicates the time point that cell differentiation occurred. In Figure 7c, at the dashed line, the cells have differentiated, and TE and PE are developed to different branches. Marker genes show significantly different patterns before and after this dashed line as shown in the figure. In Figure 7d, the first dashed line is still at the 32-cell stage, where ICM cells did not differentiate into PE and EPI cells, thus the cells belonging to both types have similar trajectory. At the second dashed line, ICM cells differentiated into PE and EPI cells, and significant differences of gene expression in the marker genes appear. The patterns of the marker cells in both Figure 7c and Figure 7d are quite consist with the cell differentiation process, which further shows that the pseudotime predicted using BLTSA can reflect the actual cell development.

## 4 Discussion and conclusion

In this work, BLTSA was proposed for pseudotime inference. This method was developed based on the local geometric structure of each cell after dimensionality reduction. BLTSA first identifies the tip and branching points, and then propagates the reliable tangent information from non-branching cells to branching cells. Global coordinates for all the single cells are determined by aligning the local coordinates based on the tangent spaces. Numerical experiments in both simulated datasets and real datasets show that BLTSA performs more robustly and gives more reasonable results compared with nine existing methods. One weakness of BLTSA is that it is developed based on the data after dimensionality reduction, and thus the results might be changed with different dimension reduction methods. Theoretically, BLAST can be used directly from the high dimensional space to one-dimensional space, but a lot of efforts are still needed to learn and explore its extension practically. Furthermore, BLTSA is not applicable to the data without manifold structure, or the data sampled heavily non-uniformly from a manifold, which is also one direction of our future work.

## Supplementary Material

btad054_Supplementary_Data

## Data Availability

The dataset Shalek and dataset Sun are available through the Gene Expression Omnibus (GEO) with accession number GSE48968 and GSE85234, respectively. The dataset Guo is available at the Mouse Genome Informatics database at http://www.informatics.jax.org/ with accession number J:140465. The dataset Petropoulos is available through ArrayExpress with accession number E-MTAB-3929. The dataset Setty is available at http://nbviewer.jupyter.org/github/dpeerlab/Palantir/blob/master/notebooks/Palantir_sample_note book.ipynb.

## References

[btad054-B1] Angerer P. et al (2016) Destiny: diffusion maps for large-scale single-cell data in R. Bioinformatics, 32, 1241–1243. 26668002 10.1093/bioinformatics/btv715

[btad054-B2] Bacher R. , Kendziorski C. (2016) Design and computational analysis of single-cell RNA-sequencing experiments. Genome Biol., 17, 1–14. 27052890 10.1186/s13059-016-0927-y PMC4823857

[btad054-B3] Bendall S.C. et al (2014) Single-cell trajectory detection uncovers progression and regulatory coordination in human b cell development. Cell, 157, 714–725. 24766814 10.1016/j.cell.2014.04.005 PMC4045247

[btad054-B4] Campbell K.R. , Yau C. (2017) Probabilistic modeling of bifurcations in single-cell gene expression data using a Bayesian mixture of factor analyzers. Wellcome Open Res., 2, 19. 28503665 10.12688/wellcomeopenres.11087.1 PMC5428745

[btad054-B5] Campbell K.R. , Yau C. (2019) A descriptive marker gene approach to single-cell pseudotime inference. Bioinformatics, 35, 28–35. 29939207 10.1093/bioinformatics/bty498 PMC6298060

[btad054-B6] Cao J. et al (2019) The single-cell transcriptional landscape of mammalian organogenesis. Nature, 566, 496–502. 30787437 10.1038/s41586-019-0969-x PMC6434952

[btad054-B7] Chen Z. et al (2019) Densitypath: an algorithm to visualize and reconstruct cell state-transition path on density landscape for single-cell RNA sequencing data. Bioinformatics, 35, 2593–2601. 30535348 10.1093/bioinformatics/bty1009

[btad054-B8] Guo G. et al (2010) Resolution of cell fate decisions revealed by single-cell gene expression analysis from zygote to blastocyst. Dev. Cell., 18, 675–685. 20412781 10.1016/j.devcel.2010.02.012

[btad054-B9] Haghverdi L. et al (2016) Diffusion pseudotime robustly reconstructs lineage branching. Nat. Methods, 13, 845–848. 27571553 10.1038/nmeth.3971

[btad054-B10] Ji Z. , Ji H. (2016) Tscan: pseudo-time reconstruction and evaluation in single-cell RNA-seq analysis. Nucleic Acids Res., 44, e117. 27179027 10.1093/nar/gkw430 PMC4994863

[btad054-B11] Kelsey G. et al (2017) Single-cell epigenomics: recording the past and predicting the future. Science, 358, 69–75. 28983045 10.1126/science.aan6826

[btad054-B12] Li L. et al (2018) Single-cell multi-omics sequencing of human early embryos. Nat. Cell Biol., 20, 847–858. 29915357 10.1038/s41556-018-0123-2

[btad054-B13] Liu S. , Trapnell C. (2016) Single-cell transcriptome sequencing: recent advances and remaining challenges. F1000Res., 5, 182. 10.12688/f1000research.7223.1 PMC4758375 26949524

[btad054-B14] Liu Z. et al (2017) Reconstructing cell cycle pseudo time-series via single-cell transcriptome data. Nat. Commun., 8, 1–9. 28630425 10.1038/s41467-017-00039-z PMC5476636

[btad054-B15] Lönnberg T. et al (2017) Single-cell RNA-seq and computational analysis using temporal mixture modelling resolves Th1/Tfh fate bifurcation in malaria. Sci. Immunol., 2, 9, eaal2192. 10.1126/sciimmunol.aal2192. PMC5365145 28345074

[btad054-B16] Mondal P.K. et al (2021) Pseudoga: cell pseudotime reconstruction based on genetic algorithm. Nucleic Acids Res., 49, 7909–7924. 34244782 10.1093/nar/gkab457 PMC8661435

[btad054-B17] Petropoulos S. et al (2016) Single-cell RNA-seq reveals lineage and X chromosome dynamics in human preimplantation embryos. Cell, 165, 1012–1026. 27062923 10.1016/j.cell.2016.03.023 PMC4868821

[btad054-B18] Qiu X. et al (2017) Reversed graph embedding resolves complex single-cell trajectories. Nat. Methods, 14, 979–982. 28825705 10.1038/nmeth.4402 PMC5764547

[btad054-B19] Saelens W. et al (2019) A comparison of single-cell trajectory inference methods. Nat. Biotechnol., 37, 547–554. 30936559 10.1038/s41587-019-0071-9

[btad054-B20] Setty M. et al (2016) Wishbone identifies bifurcating developmental trajectories from single-cell data. Nat. Biotechnol., 34, 637–645. 27136076 10.1038/nbt.3569 PMC4900897

[btad054-B21] Setty M. et al (2019) Characterization of cell fate probabilities in single-cell data with Palantir. Nat. Biotechnol., 37, 451–460. 30899105 10.1038/s41587-019-0068-4 PMC7549125

[btad054-B22] Shalek A.K. et al (2014) Single-cell RNA-seq reveals dynamic paracrine control of cellular variation. Nature, 510, 363–369. 24919153 10.1038/nature13437 PMC4193940

[btad054-B23] Shin J. et al (2015) Single-cell RNA-seq with waterfall reveals molecular Cascades underlying adult neurogenesis. Cell Stem Cell, 17, 360–372. 26299571 10.1016/j.stem.2015.07.013 PMC8638014

[btad054-B24] Street K. et al (2018) Slingshot: cell lineage and pseudotime inference for single-cell transcriptomics. BMC Genomics, 19, 1–16. 29914354 10.1186/s12864-018-4772-0 PMC6007078

[btad054-B25] Stubbington M.J.T. et al (2017) Single-cell transcriptomics to explore the immune system in health and disease. Science, 358, 58–63. 28983043 10.1126/science.aan6828 PMC5654495

[btad054-B26] Sun N. et al (2017) Inference of differentiation time for single cell transcriptomes using cell population reference data. Nat. Commun., 8, 1–12. 29187729 10.1038/s41467-017-01860-2 PMC5707349

[btad054-B27] Trapnell C. (2015) Defining cell types and states with single-cell genomics. Genome Res., 25, 1491–1498. 26430159 10.1101/gr.190595.115 PMC4579334

[btad054-B28] Trapnell C. et al (2014) The dynamics and regulators of cell fate decisions are revealed by pseudotemporal ordering of single cells. Nat. Biotechnol., 32, 381–386. 24658644 10.1038/nbt.2859 PMC4122333

[btad054-B29] Wagner D.E. et al (2018) Single-cell mapping of gene expression landscapes and lineage in the zebrafish embryo. Science, 360, 981–987. 29700229 10.1126/science.aar4362 PMC6083445

[btad054-B30] Welch J.D. et al (2016) Slicer: inferring branched, nonlinear cellular trajectories from single cell RNA-seq data. Genome Biol., 17, 1–15. 27215581 10.1186/s13059-016-0975-3 PMC4877799

[btad054-B31] Wolf F.A. et al (2019) PAGA: graph abstraction reconciles clustering with trajectory inference through a topology preserving map of single cells. Genome Biol., 20, 59–59. 30890159 10.1186/s13059-019-1663-x PMC6425583

[btad054-B32] Zhang Z. , Zha H. (2004) Principal manifolds and nonlinear dimensionality reduction via tangent space alignment. SIAM J. Sci. Comput., 26, 313–338.

[btad054-B33] Zhang Z. , Zhang X. (2021) Velosim: simulating single cell gene-expression and RNA velocity. BioRxiv.

[btad054-B34] Zhang Z. et al (2012) Adaptive manifold learning. IEEE Trans. Pattern Anal. Mach. Intell., 34, 253–265. 21670485 10.1109/TPAMI.2011.115

