# Supplementary material for: BLTSA: pseudotime prediction for single cells by branched local tangent space alignment
Source: Bioinformatics. 2023 Jan 24;39(2):btad054. doi: 10.1093/bioinformatics/btad054 (PMC9923702; doi:10.1093/bioinformatics/btad054)
Supplement: btad054_Supplementary_Data [file btad054_supplementary_data.pdf]

# Supplementary materials

## 1. BLTSA algorithm

**Algorithm 1** BLTSA: Branched Local Tangent Space Alignment

**Input:** Single-cell expression matrix $\widetilde{X} \in R^{m \times N}$.

**Output:** Pseudotime $T = [\tau_1, \cdots, \tau_N] \in R^{d \times N}$ $(d = 1)$.

1: Reduce the dimensionality to $D$ using diffusion maps and obtain a lower-dimensional data matrix $X = [x_1, \ldots, x_N] \in R^{D \times N}$;
2: Find the neighboring cells $\mathcal{N}_i$ of cell $i$, construct $X_i$;
3: Calculate $nonlinearity_i$. If $nonlinearity_i > \delta_b$, identify $i$ as a branching cell;
4: Calculate $consistency_i$. If $consistency_i > \delta_t$, identify $i$ as a tip cell;
5: Cluster non-branching cells using spectral clustering;
6: Assign all the branching cells to their nearest branch iteratively;
7: Determine local linear neighbors for all cells;
8: Calculate local coordinate in all local linear neighborhoods;
9: Align all the local tangent space and obtain the pseudotime $T = [\tau_1, \cdots, \tau_N]$.

## 2. Comparison of computational time

To give a clear picture of the computational time for all the ten methods, we implemented them on a desktop with a 2.9 GHz Processor and 16 GB memory, and recorded the computational time on dataset Setty, which consists of 4033 cells and 13367 genes. Table. 1 shows the computational time. BLTSA ranks the fifth among the ten methods.

Table 1: Computational time of all the ten methods for dataset Setty.

| Method | Ouija | SLICER | Monocle3 | MFA | DensityPath | PseudoGA | DPT | Slingshot | Palantir | BLTSA |
|---|---|---|---|---|---|---|---|---|---|---|
| Time(s) | 78,420.3 | 10,152.0 | 33.0 | 2,188.7 | 171.6 | 3,028.0 | 9.1 | 846.3 | 7.1 | 330.7 |

## 3. Robustness of the parameters

To check the robustness of the parameters, we did experiments on dataset Guo for different values of $k$, $k_{min}$ and $k_{max}$. The default values of these three parameters are $k = 50, k_{min} = 40$, and $k_{max} = 100$. We fixed two parameters, and varied the remaining one to see the results. Fig. 1 shows the results. BLTSA performs robustly for different parameters in a large range.

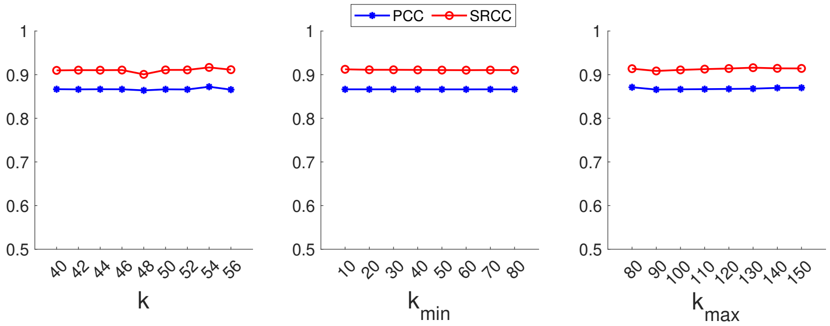


Figure 1: Results for different parameters on dataset Guo.
